# Use of aortic wall patches as leaflet replacement material during aortic valve repair

**DOI:** 10.1016/j.xjtc.2023.02.017

**Published:** 2023-04-06

**Authors:** John L. Myers, J. Brian Clark, Timothy W. James, Emily Downs, Saad M. Hasan, Robert S. Binford, Jeffrey D. McNeil, Victor M. Rodriquez, Christopher E. Mascio, Lawrence M. Wei, Vinay Badhwar, J. Scott Rankin

**Affiliations:** a Pennsylvania State University, Hershey, Pa; b St Joseph's Hospital, Tacoma, Wash; c University of Virginia, Charlottesville, Va; d Christ Hospital, Cincinnati, Ohio; e Overlake Hospital, Bellevue, Wash; f Cardiothoracic and Vascular Surgeons of Austin, Austin, Tex; g Sutter Medical Center, Sacramento, Calif; h West Virginia University, Morgantown, WVa

**Keywords:** aortic valve repair, aortic leaflet replacement, leaflet defects

## Abstract

**Objectives:**

Aortic valve repair can be limited by inadequate leaflet tissue for proper coaptation. Various kinds of pericardium have been used for cusp augmentation, but most have failed because of tissue degeneration. A more durable leaflet substitute is needed.

**Methods:**

In this report, 8 consecutive cases are presented in which autologous ascending aortic tissue was used to augment inadequate native cusps during aortic valve repair. Biologically, aortic wall is a living autologous tissue that could have exceptional durability as a leaflet substitute. Techniques for insertion are described in detail, along with procedural videos.

**Results:**

Early surgical outcomes were excellent, with no operative mortalities or complications, and all valves were competent with low valve gradients. Patient follow-up and echocardiograms to a maximum of 8 months’ postrepair remain excellent.

**Conclusions:**

Because of superior biologic characteristics, aortic wall has the potential to provide a better leaflet substitute during aortic valve repair and to expand patient categories amenable to autologous reconstruction. More experience and follow-up should be generated.

## Graphical Abstract

**Figure:**
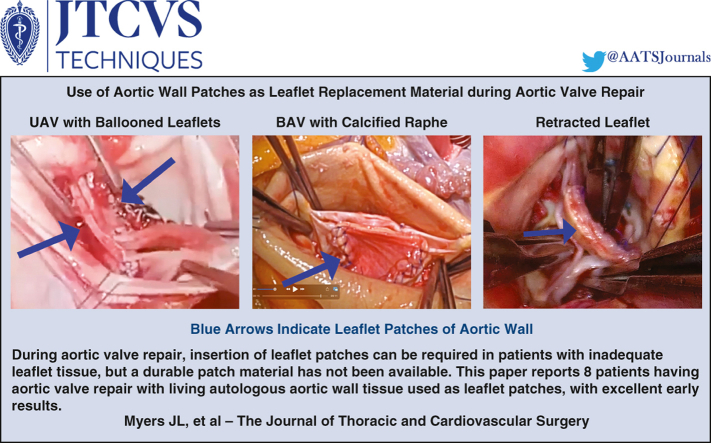

**Figure:**
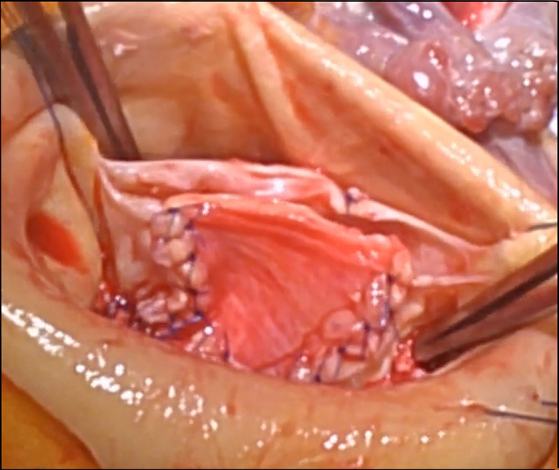
Aortic wall patching of a calcified raphe defect during bicuspid aortic valve repair.

**Central Message**

Pericardial patches for aortic leaflet replacement failed because of degeneration. The patient's aortic wall has the advantage of living autologous tissue that likely will function better long-term.

**Perspective**

During aortic valve repair, insertion of leaflet patches can be required in patients with inadequate leaflet tissue. Previous pericardial patches tended to degenerate and fail, and a good patch material has not been available. The paper reports 8 patients having aortic valve repair with living autologous aortic wall tissue used as leaflet patches, with excellent early results.

In 1959, Dr Charles P. Bailey first reported using ascending aortic wall as a leaflet replacement material during aortic valve repair (AVr).^1^ He described 25 cases of aortic valve reconstruction for aortic insufficiency (AI), with success in 18. Several of the patients had creation of aortic valve cusps using aortic wall, although exact details and follow-up were not documented. Bailey’s pioneering work, however, was largely forgotten. Subsequent creation of aortic valve leaflets from Teflon, fascia lata, and pericardium^2^^,^^3^ proved unsuccessful, and practice migrated toward replacing cardiac valves with artificial prostheses. Subsequent definition of excessive prosthetic valve–related complications^4^ rekindled interest in cardiac valve repair,^5^ which now dominates mitral and tricuspid valve practice.^6^ AVr also is becoming more common for insufficient aortic valves.^7^, ^8^, ^9^, ^10^, ^11^, ^12^ When adequate native valve leaflets exist, the current success of AVr is excellent^13^; however, in patients with defective leaflet tissue, prosthetic valve replacement still is required. Pericardial leaflet patches have been thoroughly tested, but have almost uniformly failed.^14^ Availability of an adequate leaflet replacement material would be a major facilitator for AVr. The goal of this report is to reassess the novel concept of using aortic wall as a living autologous leaflet replacement material during AVr.

## Methods

In the course of routine AVr practice, 8 patients were found to have inadequate leaflet tissue to achieve successful coaptation. In each patient consecutively, autologous ascending aortic tissue was used to fashion leaflet augmentation patches, with full success in all. In each case, standard echocardiography and video recordings of the procedures were obtained for educational purposes. Brief summaries of the procedures are as follows.

### Case Presentations

#### Patient 1: unicuspid valve with leaflet tears from balloon valvuloplasty

**Figure 1:**
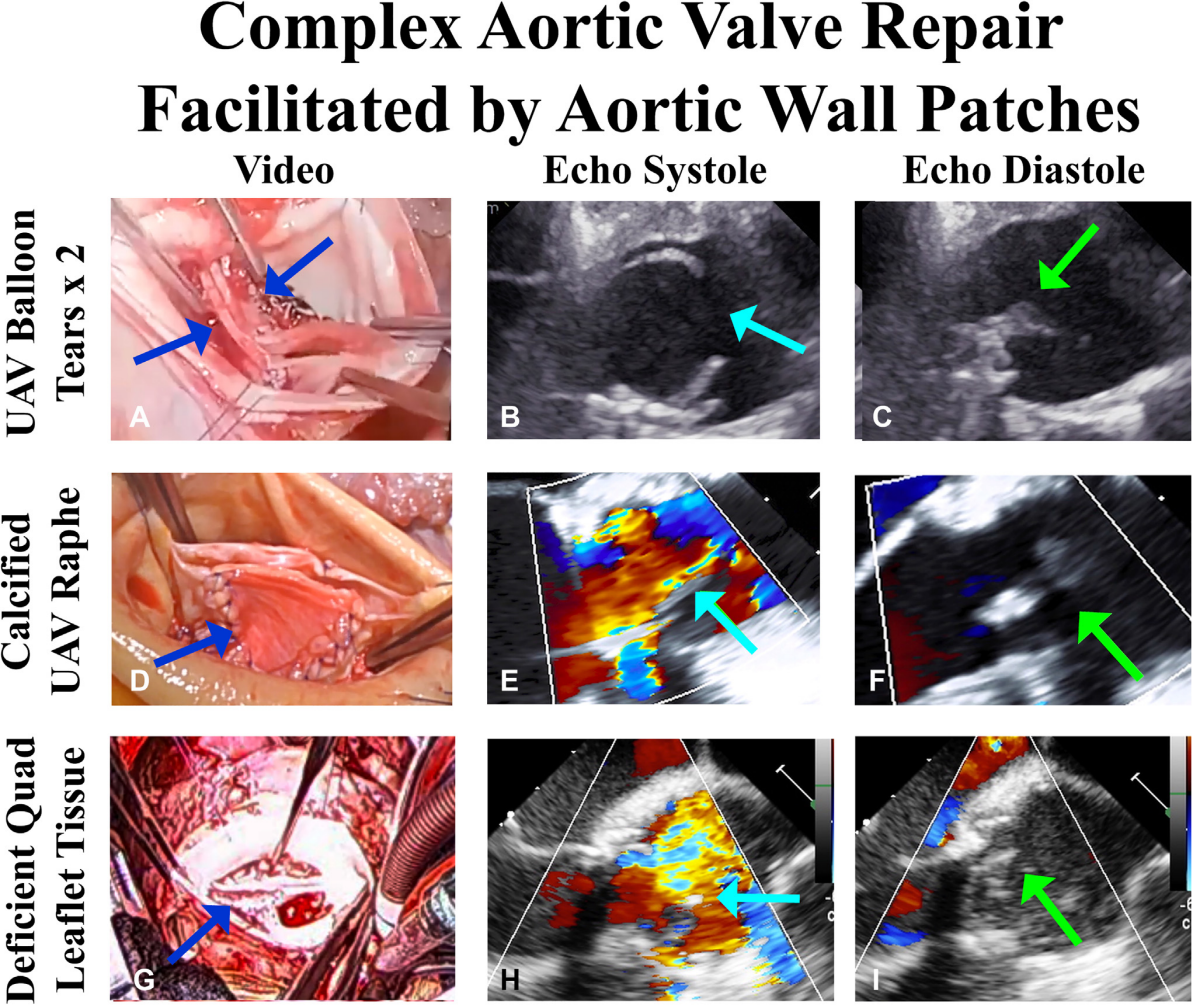
Illustrations from patients 1, 2, and 4 receiving aortic wall patches during complex aortic valve repair. The first patient had a torn *right*-/noncoronary commissure from balloon valvuloplasty of a unicuspid valve in childhood (A), with inadequate fused and nonfused cusp tissue for repair. Patches of ascending aorta were placed into both leaflets, using interrupted 6-0 PROLENE sutures (Ethicon; *dark blue arrows*). After repair, the leaflets opened well (B), with excellent coaptation height (C). In the second unicuspid patient, the repair was started with a *right*-/noncoronary commissurotomy, but the *left*-/*right* coronary raphe contained a broad transmural mass of calcium. The calcified raphe and cleft tissue were resected and replaced with a patch of aortic wall tissue (D, *dark blue arrow*). Postrepair, the valve opened and coapted well (E and F). The third patient had a quadricuspid truncus valve with a deficient left coronary leaflet. The defect was patched with aortic wall (G), and after repair, valve opening and coaptation were excellent (H and I). *UAV*, Unicuspid valve; *Quad*, quadricuspid valve. *Light blue arrows* show the leaflets open, and *green arrows* depict the closed, coapted leaflets.

The first case was performed on June 8, 2022. The patient was a 16-year-old male born with an obstructed unicuspid aortic valve. He underwent aortic balloon valvuloplasty in childhood, relieving the obstruction, but inducing AI (Video 1). He presented currently with severe AI, New York Heart Association class III congestive heart failure, and deteriorating left ventricular function. The ascending aorta was 4.3 cm in diameter. At surgery, he was found to have a major fusion and cleft of the right-/left-coronary commissure and a torn right-/noncoronary commissure with inadequate leaflet tissue in that area.^15^ Both leaflets were patched with ascending aortic tissue (Figure 1, *A-C*). The valve was sized to a 23-mm annuloplasty ring, with free-edge length (FEL) of both leaflets needing to be half of ring circumference, or 36 mm. Thus, 2 aortic wall patches reconstituted each leaflet to 36 mm FEL. After placement of the annuloplasty ring, the patches were inserted, using interrupted sutures of 6-0 PROLENE (Ethicon). The ascending aorta was replaced with a 30-mm Dacron graft. After repair, the valve was fully competent with good leaflet motion and a mean valve gradient of 6 mm Hg. At 10 months of follow-up, the patient was fully functional with continued excellent valve performance by repeat echocardiography.

#### Patient 2: unicuspid valve with severely calcified raphe and cleft

This patient was a 67-year-old female presenting with mild heart failure, severe AI, and a 47-mm ascending aorta. The aortic valve also was unicuspid (Video 2), and the right-/left-coronary fused raphe exhibited severe broad transmural calcification, too extensive for ultrasonic debridement.^13^ The calcified raphe was resected, a 23-mm annuloplasty ring was placed, and a funnel-shaped patch of aortic wall was sutured into the raphe defect using 6-0 PROLENE (Ethicon) interrupted sutures. Both leaflets were adjusted with plication stitches to a target FEL of 36 mm (Figure 1, *D-F*), and the ascending aorta was replaced with a 30-mm Dacron graft. After repair, both leaflets moved well, with full valve competence, and a mean gradient of 5 mm Hg. The patient recovered uneventfully, and echocardiographic valve function remained excellent in follow-up.

#### Patient 3: bicuspid valve with severely calcified raphe and cleft

The patient was a 48-year-old asymptomatic male with a 45-mm ascending aorta discovered on a computed tomography scan for coronary screening. Echo showed grade 3 AI, a calcified bicuspid aortic valve (BAV), and normal left ventricular function. At surgery, the valve had a Sievers type 1 right-left bicuspid fusion, the annulus was 27 mm, and the valve sized to a 23-mm bicuspid ring. The fused leaflet raphe contained a broad transmural mass of calcium, which was resected. A 23-mm bicuspid ring was placed, and the fused leaflet defect was patched with ascending aorta. Because the procedure was similar to patient 2, a video was not produced. After repair, the valve was completely competent, with a mean valve gradient of 14 mm Hg. The patient recovered uneventfully and was discharged home on the fourth postoperative day. At follow-up, his clinical status and valve function remained excellent.

#### Patient 4: quadricuspid truncus valve with hypoplastic left-coronary cusp

The patient was an 8-year-old male born with truncus arteriosus type II, interrupted aortic arch type A, and secundum atrial septal defect. He had surgery in infancy, consisting of ventricular septal defect patch closure, atrial septal defect closure, arch reconstruction, and an 11-mm right ventricle to pulmonary artery conduit. Recently, he developed exercise intolerance and easy fatiguability. Echocardiography showed mild aortic stenosis, moderately severe AI, flow reversal in the descending aorta, left ventricular dilation, moderate conduit stenosis, severe conduit valve insufficiency, and moderate right ventricular enlargement. The aortic annulus was 23 mm (Video 3), and the 2 rightward leaflets were joined with a fusion. The leftward noncoronary leaflet was good-sized, and the left-coronary leaflet was small. The rightward leaflet sized to a 21-mm sizer, so a 21-mm bicuspid ring was sutured into the 2 larger commissures and sinuses. The noncoronary and small left-coronary cusps were sutured together with interrupted 6-0 PROLENE (Ethicon) sutures, but the left coronary component was too short for full coaptation. Therefore, a small patch of aortic wall was excised from the distal aortotomy and sutured into the left-coronary leaflet defect using 7-0 PROLENE (Ethicon) interrupted sutures (Figure 1, G-I). The right-sided conduit was changed to a 22-mm homograft, and after repair, the AI was trivial to mild with a mean valve gradient of 11 mm Hg (Video 3). The patient was discharged home at 7 days, and recovery was uneventful, with stable late echos.

#### Patient 5: intermediate-type BAV with hypoplastic left coronary cusp and thin cleft tissue

The patient was a 59-year-old male who presented with mild-to-moderate heart failure and palpitations. Echocardiography revealed a severe posterior AI jet, thickened leaflets, and an ejection fraction of 0.45 (Video 4). New-onset atrial fibrillation was present. He underwent a bi-atrial maze procedure and AVr. The aortic valve was an intermediate-type BAV,^16^ with a small left cusp. The noncoronary cusp sized to a 23-mm bicuspid annuloplasty ring, and the annulus was 27 mm. A 23-mm bicuspid ring was implanted across the noncoronary commissures, and the cleft in the left-right fused leaflet was closed. The cleft tissue was very thin, and part of the cleft was closed using four 6-0 PROLENE (Ethicon) horizontal mattress sutures supported by reinforcing strips of native aortic wall. The FEL of the noncoronary cusp was shortened using 6-0 PROLENE (Ethicon) plication sutures down to 36 mm, or half of a 23-mm ring circumference. Postrepair echo showed no AI and a transvalvular mean gradient of 8 mm Hg (Video 4). He was discharged in sinus rhythm on the 11th postoperative day, and the valve continued to be fully competent at follow-up.

#### Patient 6: multileaflet prolapse with ruptured right-coronary fenestration

This patient was a 62-year-old male who presented with heart failure and was found to have a dilated annulus and severe posterior AI jet (Video 5), consistent with right coronary leaflet prolapse.^17^ Annular diameter was 25 mm, and coronary angiography showed 2-vessel coronary disease. On closer inspection of the echo, the right leaflet was flail, and the jet actually originated more from the right-left commissure, suggesting a commissural defect. At surgery, a large fenestration in the right-coronary commissure had ruptured, causing severe prolapse of that leaflet. The leaflets sized to a 21 mm trileaflet annuloplasty ring which was implanted.^13^ The right cusp continued to prolapse because of the ruptured fenestration, so a 4-mm strip of ascending aorta was harvested from the aortotomy and sutured into the fenestration defect using 6-0 PROLENE (Ethicon) interrupted sutures. The left-coronary cusp also was plicated, and at the end, all 3 leaflets had equivalent effective heights. After bypass, the valve leaflets moved well with good coaptation height, only trivial AI, and a 10 mm Hg mean gradient. The aortotomy was closed primarily, and 2 coronary bypasses were constructed. The patient did well, and the valve remained competent at follow-up echo.

#### Patient 7: scarred and retracted non- and left coronary leaflets

The patient was a 53-year-old male who presented with chest pain, an 80% proximal left anterior descending coronary stenosis, and severe AI. Echocardiography showed a trileaflet aortic valve, severe central leak, and normal aortic dimensions. A coronary graft was placed. The valve was small (Video 6), with a 23-mm annulus, significant retraction of the noncoronary leaflet, and mild retraction of the left. The leaflets sized to a 19-mm annuloplasty ring, which was implanted.^13^ The retracted noncoronary cusp limited coaptation, and a 15-mm by 7-mm patch of ascending aorta was harvested from the distal aortotomy and sutured to the central leaflet with 6-0 PROLENE interrupted sutures (Ethicon). The patch also seemed to coapt well to the mild left-coronary leaflet retraction (Video 6). After bypass, AI was trivial with a mean gradient of 7 mm Hg. The patient recovered uneventfully and was discharged home on the third postoperative day. Follow-up echocardiography showed continued excellent aortic valve function.

#### Patient 8: infectious endocarditis

The patient was a 39-year-old female with diabetes who presented with respiratory failure and acute methicillin-resistant *Staphylococcus aureus* septicemia requiring endotracheal intubation. Initial transthoracic echo was negative. After several weeks of intravenous daptomycin, she recovered, and blood cultures turned negative. Transesophageal echo showed grade 3 to 4 AI with thickened aortic leaflets (Video 7). Operatively, a hole was present in the central noncoronary cusp, but the other leaflets looked good. The annulus was normal in diameter at 19 mm, so annuloplasty was not performed. A 10 × 10-mm aortic wall patch was sutured to the hole with interrupted 6-0 PROLENE sutures (Ethicon), leaving the free-edge 2 mm taller than the leaflets. The patch was trimmed, and then the 3 leaflets met nicely in the midline with good coaptation. The aortotomy, including the patch defect, was closed primarily. After repair, the patched leaflet opened well with no residual leak and an 8 mm Hg mean pressure gradient. The patient recovered uneventfully, with continued good valve function, and is currently 6 months’ postoperative.

The study was approved by the institutional review board of West Virginia University for retrospective analysis of deidentified clinical data (#2005016064; Approval date May 29, 2020; expiration date May 28, 2025). Informed consent was obtained preoperatively by the operating surgeon from every patient.

## Results

Individual data for the 8 patients are presented in Table 1. A broad spectrum of ages and lesions existed, including 2 pediatric patients and several with complex forms of BAV disease. Two were reoperations, and 6 were male. Most were symptomatic and had LV dysfunction. All patients had grade 3 to 4 AI preoperatively, and each achieved grade 0 AI after repair (*P* < .0001 by 2-tailed paired *t*-test). Patches were obtained from the resected ascending aorta in 3 patients, and from the aortotomy rim (with primary closure) in the rest. Average ring size was 22.1 mm, and mean valve gradients were uniformly low (average 8.4 mm Hg). There were no operative mortalities or major complications, and all patients recovered uneventfully. Early clinical and echocardiographic follow-up data were excellent to a maximum of 10 months (mean 7.8 months). All patients currently are being followed on a regular basis.

**Table 1:** Clinical characteristics of patients undergoing leaflet patches of aortic wall

Patient no.	Age, y	Sex, M/F	Diagnosis	Reoperation, Y/N	NYHA class 1-4	LVEF	Asc. Ao. Dil.	Pre-AI Gr	Post-AI Gr	Post-mean gradient	Ring size, mm	F/U time, mo
1	16	M	UAV	Y	3	0.25	Y	4	0	6	23	8
2	67	F	UAV	N	2	0.35	Y	4	0	5	25	7
3	48	M	BAV	N	1	0.60	Y	3	0	14	23	6
4	8	M	QAV	Y	3	0.45	N	4	0	11	21	6
5	59	M	IBAV	N	3	0.45	N	4	0	8	23	6
6	62	M	Fen.	N	4	0.60	N	4	0	10	21	5
7	53	M	NR	N	4	0.55	N	4	0	7	19	5
8	39	F	IE	N	4	0.45	N	3	0	8	N	4
Mean	44.0	75%	–	25%	3.0	0.46	38%	3.8	0	8.4	22.1	7.8
SD	21.6	M	–	Y	1.1	0.12	Y	0.5	0	2.9	2.0	1.5

Gradients are in mm Hg. *M*, Male; *F*, female; *Y*, yes; *N*, no; *NYHA*, New York Heart Association; *LVEF*, left ventricular ejection fraction; *Asc. Ao. Dil.*, ascending aortic dilatation; *AI*, aortic insufficiency, *Gr*, grade, *F/U*, follow-up; *UAV*, unicuspid valve, *BAV*, bicuspid valve; *QAV*, quadricuspid valve; *IBAV*, intermediate-type bicuspid valve; *Fen.*, fenestration; *NR*, nodular retraction; *IE*, infectious endocarditis; *SD*, standard deviation.

## Discussion

Although 90% to 95% of pure AI valves can be repaired with geometric ring annuloplasty using native valve leaflets alone,^13^ a few pathologies have inadequate cusp tissue for coaptation. Addition of patch material to the leaflets is the repair solution, but previous experiences with pericardial patches for this purpose were fraught with a high incidence of tissue degeneration,^14^ no matter the material. The development of aortic wall leaflet patches could assist with this problem, and this approach has the potential to increase AI repair rates toward 100%.

The biologic advantages of autologous aortic wall for the purpose of AVr patches are several. Autologous aorta is readily available. The aortic wall receives its nutrition primarily from luminal diffusion and should remain a living vascular tissue after implantation. The tissue is immunologically autologous and might not scar, calcify, or degenerate. Aortic wall structure is highly fibroelastic and accustomed to systemic pressure; thus, it could function well long term. Aortic tissue handles well and is easy to suture. Ascending aorta can be replaced with Dacron graft material, with a definable rate of late consequences. Because AVr is performed through a proximal aortotomy, only an extra 15 minutes for the distal aortic suture line is required, even if the entire ascending aorta is harvested. Suturing the patches adds another 15 to 20 minutes, and although these steps do increase procedural complexity, clamp times of 2 to 3 hours are well-tolerated with current myocardial protection, with demonstrated operative mortality and complication rates of near-zero for the most complex root procedures.^9^^,^^10^ As in this study, greater-risk patients can be operated with consistent and standardized outcomes.

In these patients, small patches could be excised from the distal aortotomy, and the aorta closed primarily, adding little to the procedures. Although the aortic wall is thicker than normal leaflets, it is no thicker than some types of pericardium that have been employed.^18^ The tissue is tough and holds sutures well. In this series, aortic patches seemed to move well in vivo with low-valve gradients. When depressurized, the normally concave endothelial surface of the aorta became convex (perhaps because of transmural elastic gradients), making it an ideal shape for leaflet replacement. It is possible that coaptation with other leaflets could take stress off the aortic patches, and thus, be accompanied by minimal structural alteration over time.

In this series, aortic wall patches were used in many ways. In patient 1, 2 large leaflet defects were patched with good success. The understanding that repaired leaflet FEL should be half of annuloplasty ring circumference provides an objective reference for patch size. Defects caused by resection of severely calcified raphe tissues in patients 2 and 3 were replaced with funnel-shaped patches to facilitate fused leaflet opening. In patient 4, with truncus arteriosus, congenitally deficient leaflet tissue could be augmented to provide adequate surface area for coaptation. Deficient commissures could be bridged for ruptured fenestrations or detached commissures, a problem that has vexed AVr for a while.^17^ In patient 7, a centrally retracted leaflet could be addressed by insertion of an “artificial nodulus Arantius” to allow central leaflet coaptation. In this case with 2 leaflets retracted, augmenting only one with a larger patch provided enough tissue for full valve competence. Finally, infectious endocarditis in patient 8 was simply repaired by insertion of a single leaflet patch. As with many patients with endocarditis, the annulus was normal, so annuloplasty was unnecessary. This approach to endocarditis could be appealing to minimize operative time and inserted foreign body.

All leaflet suture lines were constructed from interrupted 6-0 or 7-0 PROLENE suture (Ethicon) to avoid leaflet distortion associated with running technique. The intima faced coaptation, with knots toward the sinuses. When the patch had to be attached to the aorta, in a greater stress region like the commissural top, reinforcement with fine Dacron pledgets provided additional support. In early follow-up, this approach seemed very stable with no disruptions, which may be due in part to the inherent strength of aortic tissue. This repair series is an achievement, since each of these patients would have received a prosthetic valve replacement 1 year ago. Also noteworthy is the fact that all patients had grade 3 to 4 AI before repair, which uniformly fell to Grade zero, associated with low-valve gradients. This finding supports the principle that adding leaflet surface area increases efficacy in both aspects of valve function. Although pulmonary artery also could be used for this purpose,^19^, ^20^, ^21^ our bias is that aortic wall has the strength and tissue characteristics to function better long-term in the systemic circulation.

Finally, some speculation might be in order. If ascending aortic wall performs well as aortic leaflet patches, this tissue potentially could be extended to autologous trileaflet aortic valve replacement. The shape for constructing aortic valve leaflets is well-understood from computed tomography angiographic studies,^22^^,^^23^ and ample ascending aorta usually is available to fashion 3 leaflets. This approach would be less destructive than excising the normal pulmonary valve for Ross procedures,^24^ and would obviate long-term problems with pulmonary conduit dysfunction. The 3 aortic resection patients in this series had only mild aortic dilatation (43, 45, 47 mm) associated with bicuspid disease, with normal gross appearance; thus, these aortic patches should function well without distortion or stretching. However, the use of aortic wall in more severe aortopathies, such as Loeys–Dietz syndrome, might be less appealing, although the 3-dimensional stress characteristics of centrally coapted leaflets is low,^25^ and one could hypothesize that stresses imposed on an aortic leaflet patch would be less than experienced in situ with aortic distension by arterial blood pressure.

In performing standard Ozaki procedures,^26^ it sometimes has been difficult to size leaflets based on intercommissural chord lengths, especially with different commissural heights, sinus sizes, and/or leaflet areas.^18^ When using aortic wall, we would favor performing bileaflet or trileaflet replacement in combination with ring annuloplasty in order to provide a “normal” or consistent annular geometric “stent” for all of the sinuses. Then, the same leaflet design could be used for all cusps, simplifying the procedure, stabilizing the annulus, and standardizing the operation. Finally, aortic leaflets possibly could be used for fabricating right ventricular to pulmonary artery conduits, theoretically creating a more autologous and durable valve that could last better than current homografts.

**Figure 2:**
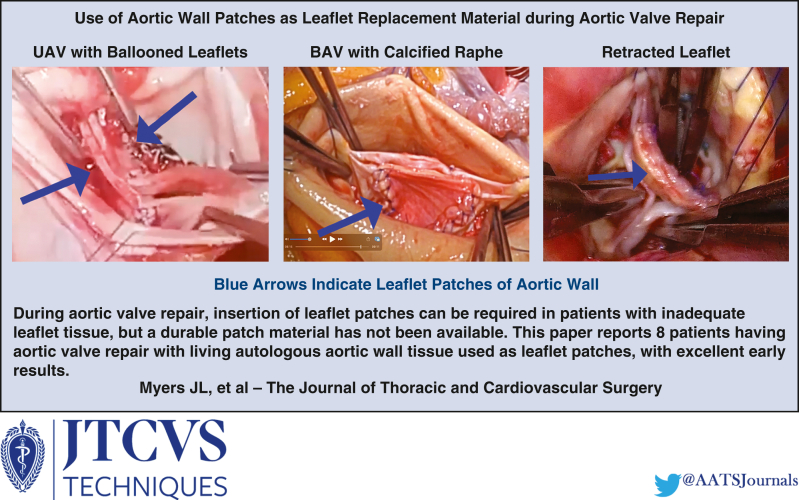
Graphical abstract. *UAV*, Unicuspid valve; *BAV*, bicuspid aortic valve.

In summary, AI sometimes is associated with inadequate leaflet tissue that limits valve coaptation. Pericardial leaflet extension for this purpose has experienced a high incidence of tissue degeneration and failure. Initial results using ascending aortic wall as a leaflet substitute have been excellent, allowing reconstruction of a broad spectrum of leaflet defects. More experience and follow-up will be necessary to fully assess this approach, but aortic wall patches could increase AI repair rates, as well as extend AVr into areas not currently encompassed (Figure 2).

### Conflict of Interest Statement

The hemispherical aortic annular reconstructive technology (HAART) aortic annuloplasty rings were developed by BioStable Science and Engineering, Austin, Texas (www.biostable-s-e.com) and are approved in the United States by the Food and Drug Administration (21 CFR 870.3800) and CE Marked in Europe (G7 103732 0008). Dr Rankin is a consultant for this company. All other authors reported no conflicts of interest.

The *Journal* policy requires editors and reviewers to disclose conflicts of interest and to decline handling or reviewing manuscripts for which they may have a conflict of interest. The editors and reviewers of this article have no conflicts of interest.

## Supplementary Data

Video 1 Aortic wall as a leaflet replacement material during repair of unicuspid valve with torn leaflets. Video available at: https://www.jtcvs.org/article/S2666-2507(23)00111-6/fulltext.

Video 2 Using a patch of ascending aortic wall to replace a severely calcified raphe and cleft during unicuspid valve repair. Video available at: https://www.jtcvs.org/article/S2666-2507(23)00111-6/fulltext.

Video 3 Repair of a quadricuspid truncus valve with inadequate leaflet tissue using a leaflet patch of aortic wall. Video available at: https://www.jtcvs.org/article/S2666-2507(23)00111-6/fulltext.

Video 4 Two-leaflet repair of an intermediate-type bicuspid aortic valve with very thin cusp tissue. Video available at: https://www.jtcvs.org/article/S2666-2507(23)00111-6/fulltext.

Video 5 Repair of a ruptured aortic leaflet fenestration using a patch of aortic wall. Video available at: https://www.jtcvs.org/article/S2666-2507(23)00111-6/fulltext.

Video 6 Aortic valve repair for leaflet retraction using an augmentation patch of aortic wall. Video available at: https://www.jtcvs.org/article/S2666-2507(23)00111-6/fulltext.

Video 7 Use of an aortic wall patch to repair an endocarditic leaflet hole. Video available at: https://www.jtcvs.org/article/S2666-2507(23)00111-6/fulltext.
