# Supplementary figures and images for: Use of aortic wall patches as leaflet replacement material during aortic valve repair
Source: JTCVS Tech. 2023 Apr 6;19:30–7. doi: 10.1016/j.xjtc.2023.02.017 (PMC10268232; doi:10.1016/j.xjtc.2023.02.017)

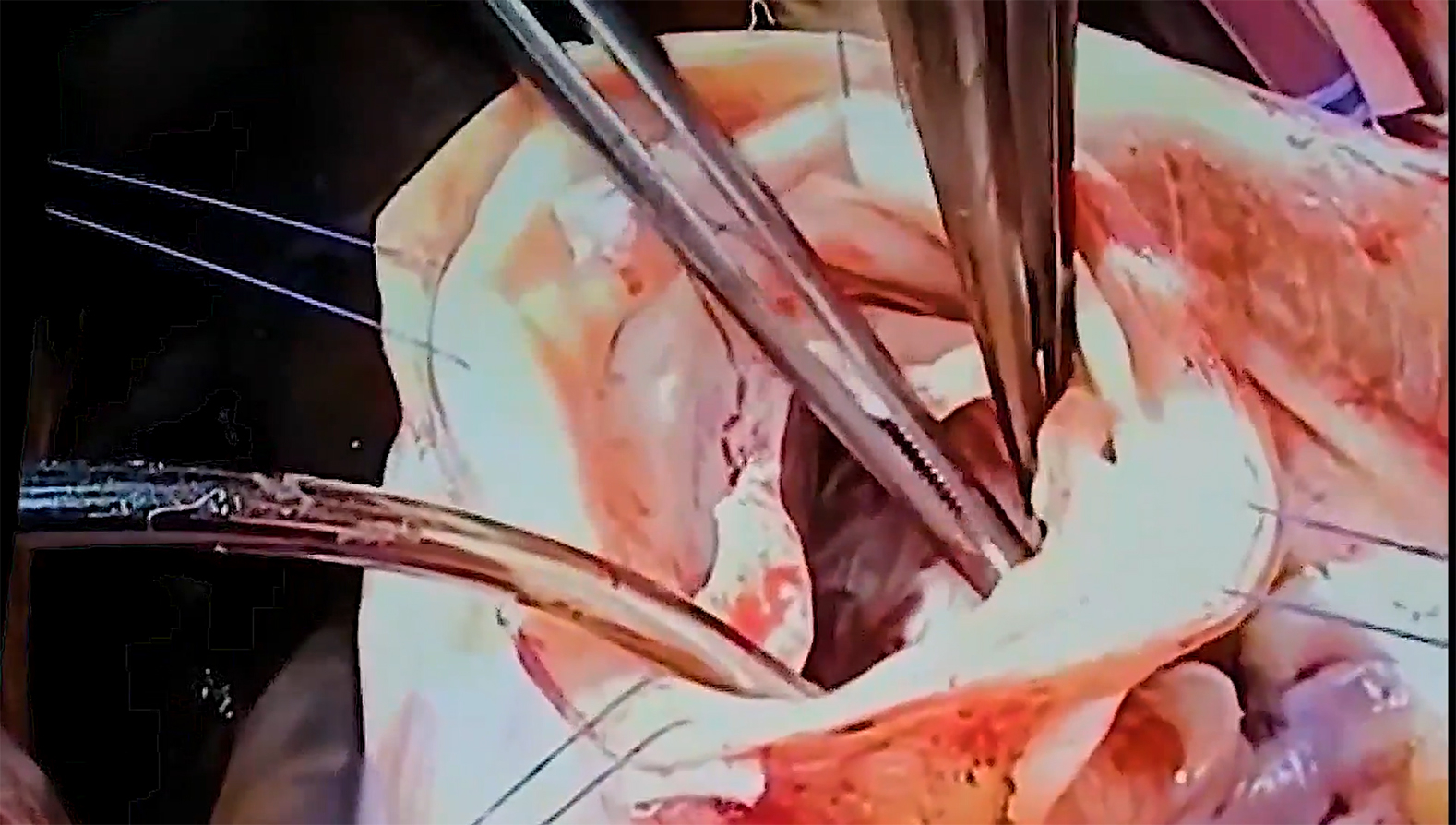

Supplement: Video 1 — Aortic wall as a leaflet replacement material during repair of unicuspid valve with torn leaflets. Video available at: https://www.jtcvs.org/article/S2666-2507(23)00111-6/fulltext. [file fx3.jpg]

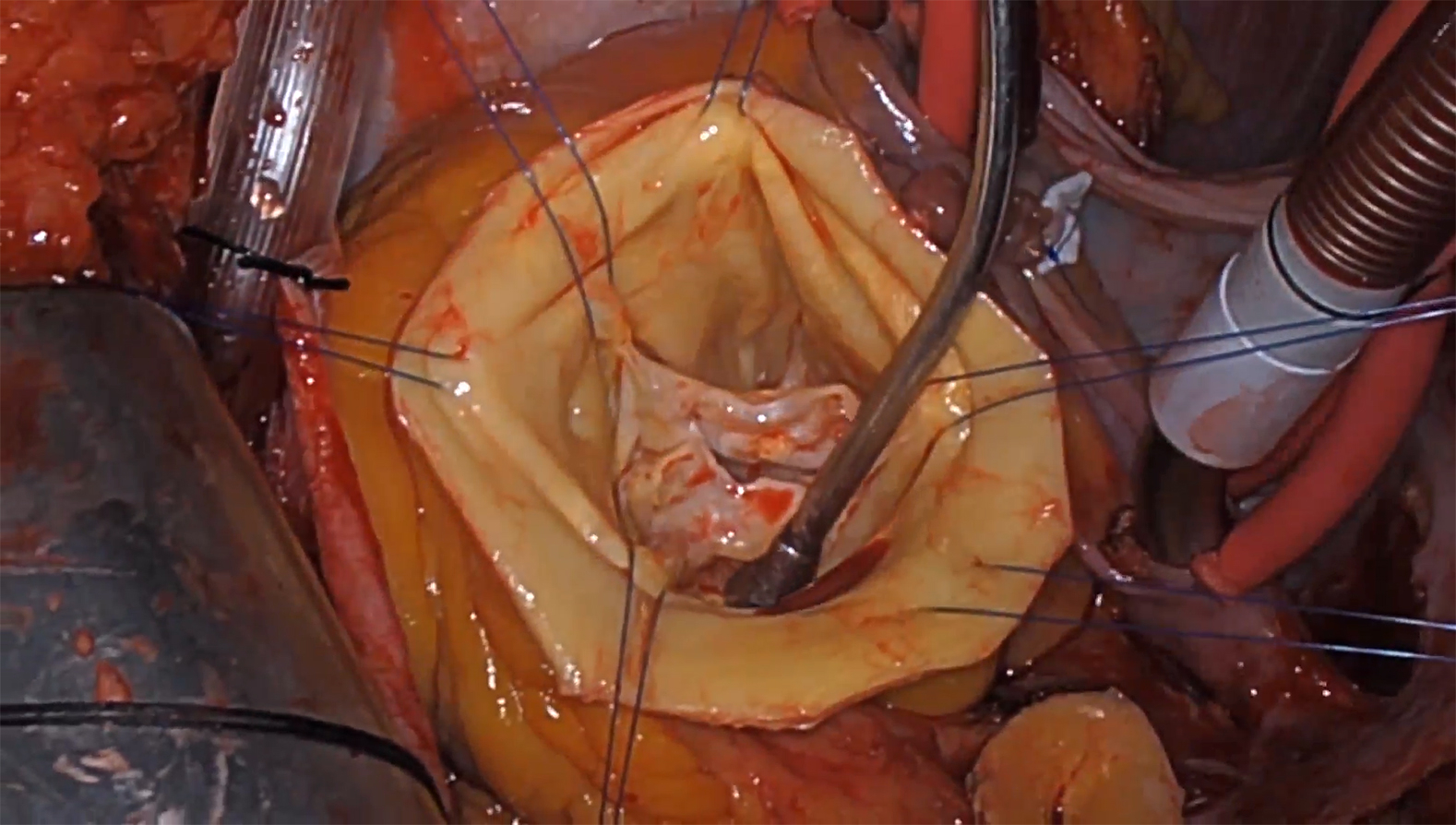

Supplement: Video 2 — Using a patch of ascending aortic wall to replace a severely calcified raphe and cleft during unicuspid valve repair. Video available at: https://www.jtcvs.org/article/S2666-2507(23)00111-6/fulltext. [file fx4.jpg]

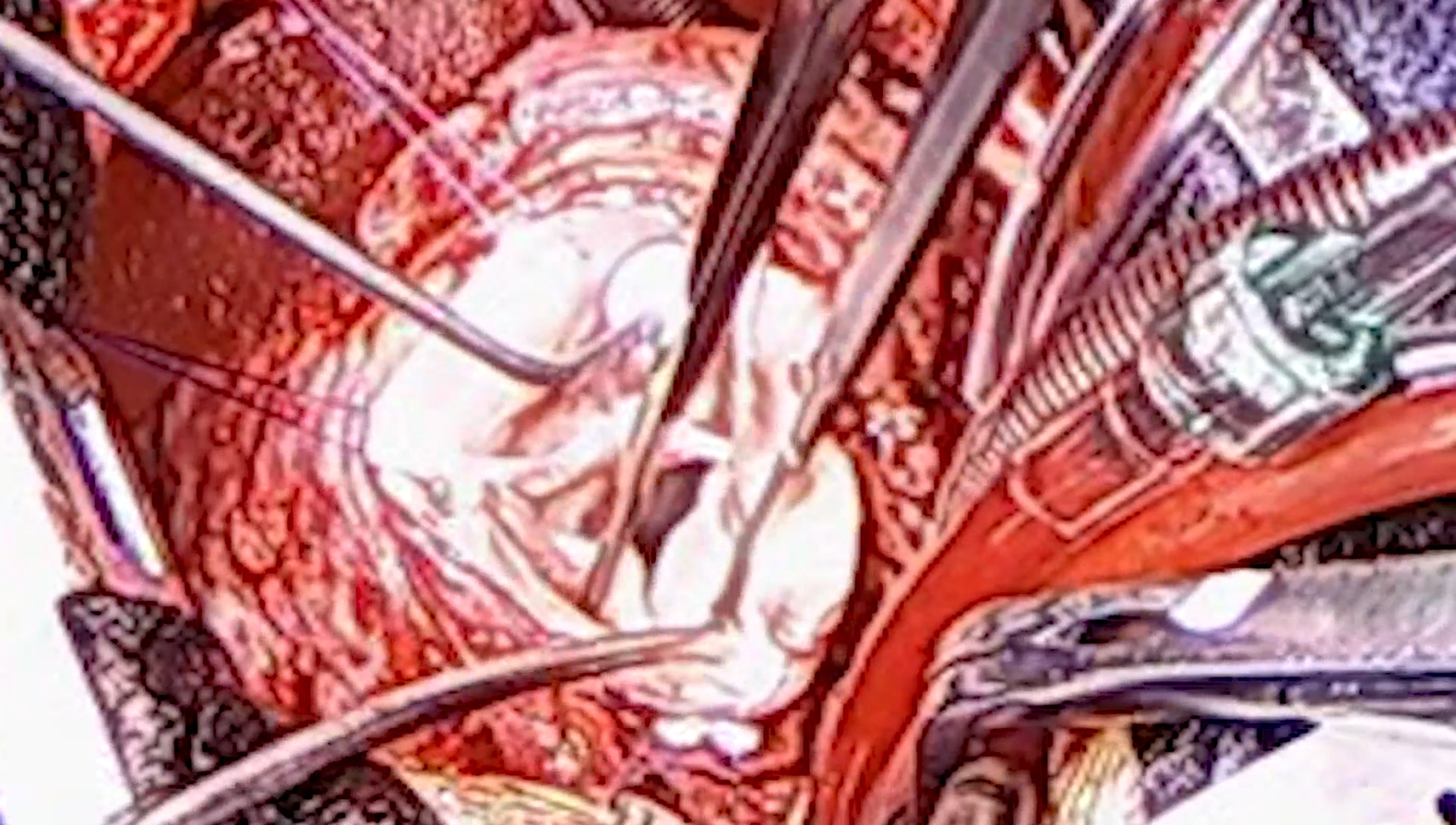

Supplement: Video 3 — Repair of a quadricuspid truncus valve with inadequate leaflet tissue using a leaflet patch of aortic wall. Video available at: https://www.jtcvs.org/article/S2666-2507(23)00111-6/fulltext. [file fx5.jpg]

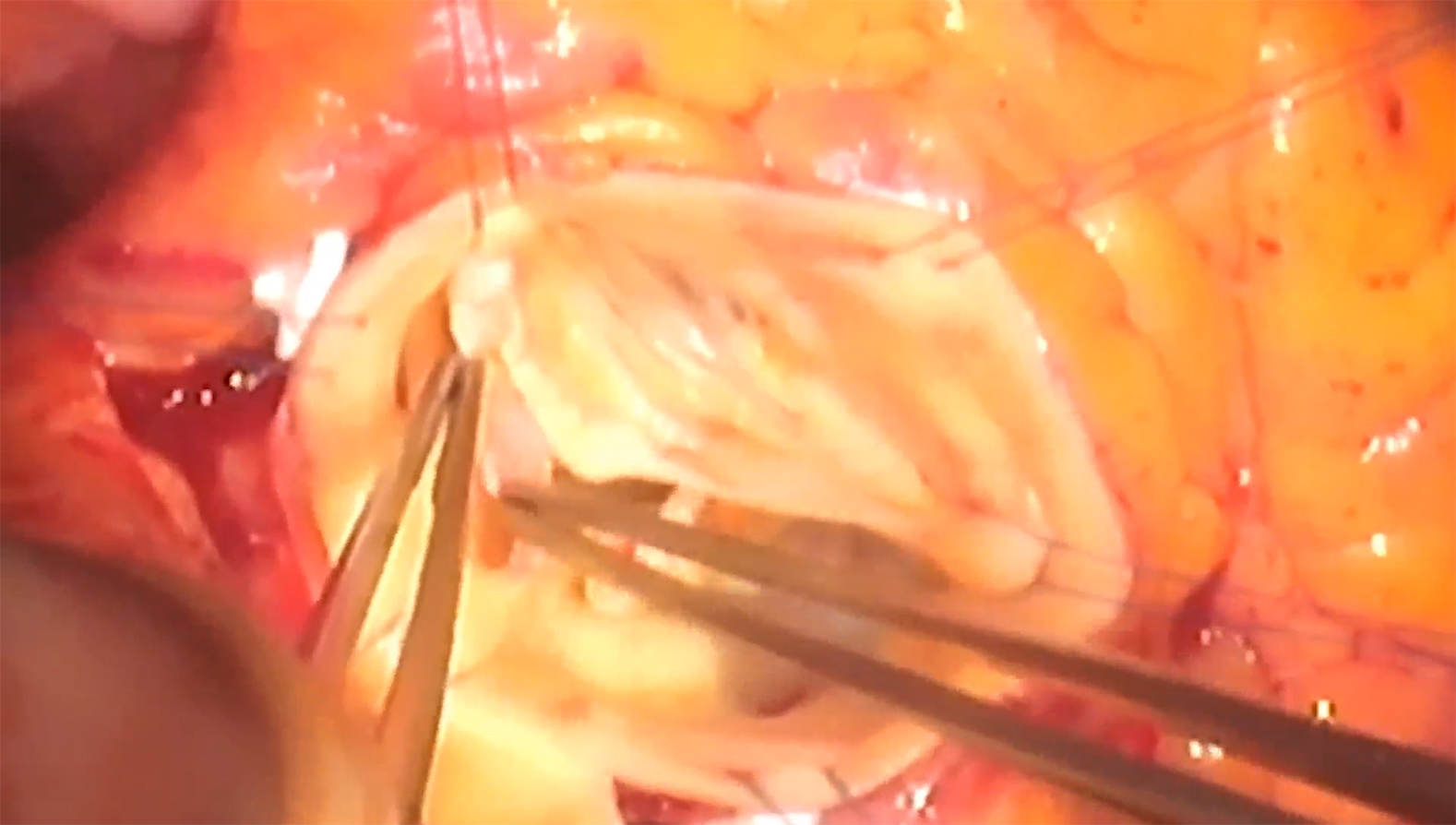

Supplement: Video 4 — Two-leaflet repair of an intermediate-type bicuspid aortic valve with very thin cusp tissue. Video available at: https://www.jtcvs.org/article/S2666-2507(23)00111-6/fulltext. [file fx6.jpg]

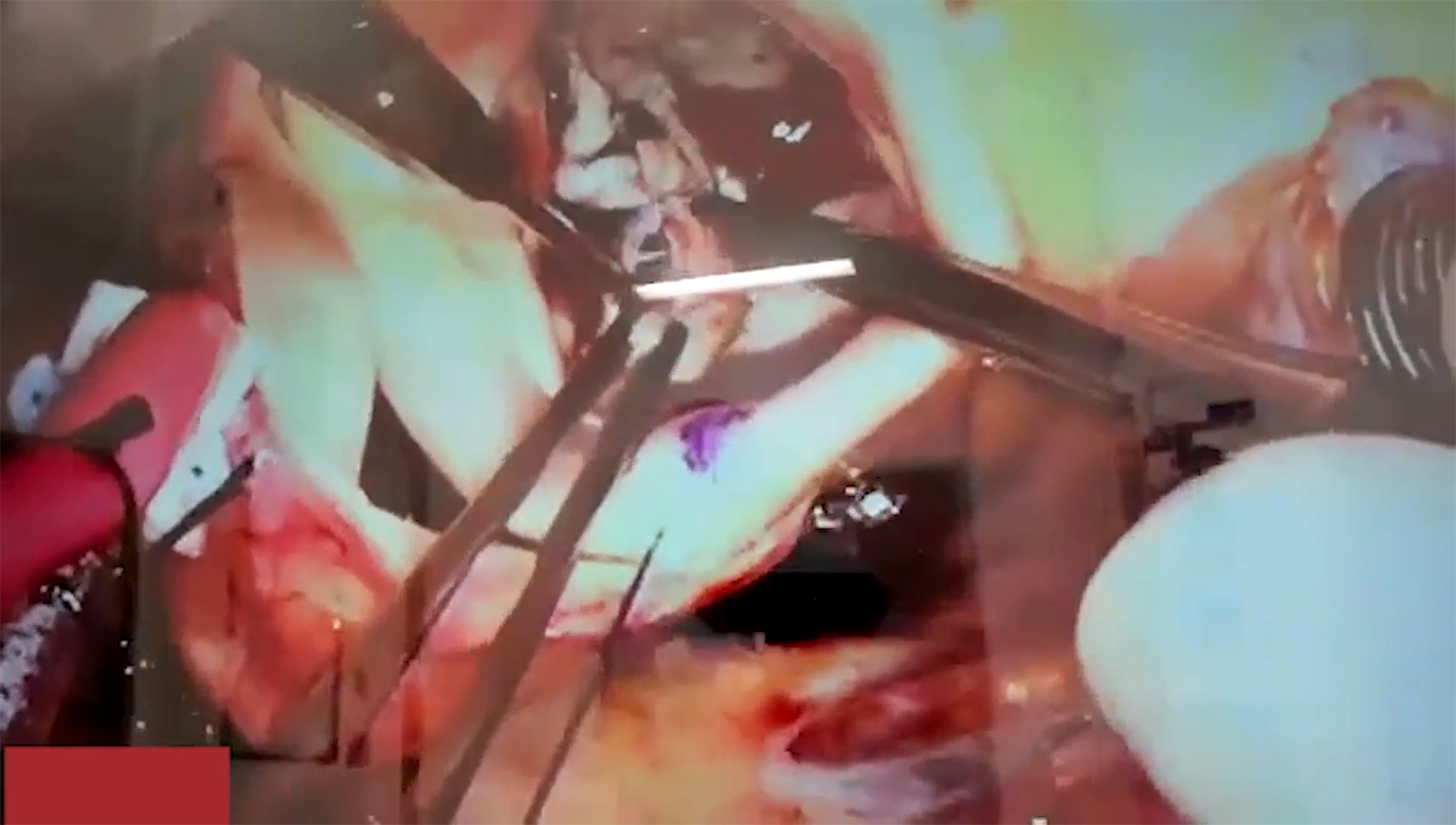

Supplement: Video 5 — Repair of a ruptured aortic leaflet fenestration using a patch of aortic wall. Video available at: https://www.jtcvs.org/article/S2666-2507(23)00111-6/fulltext. [file fx7.jpg]

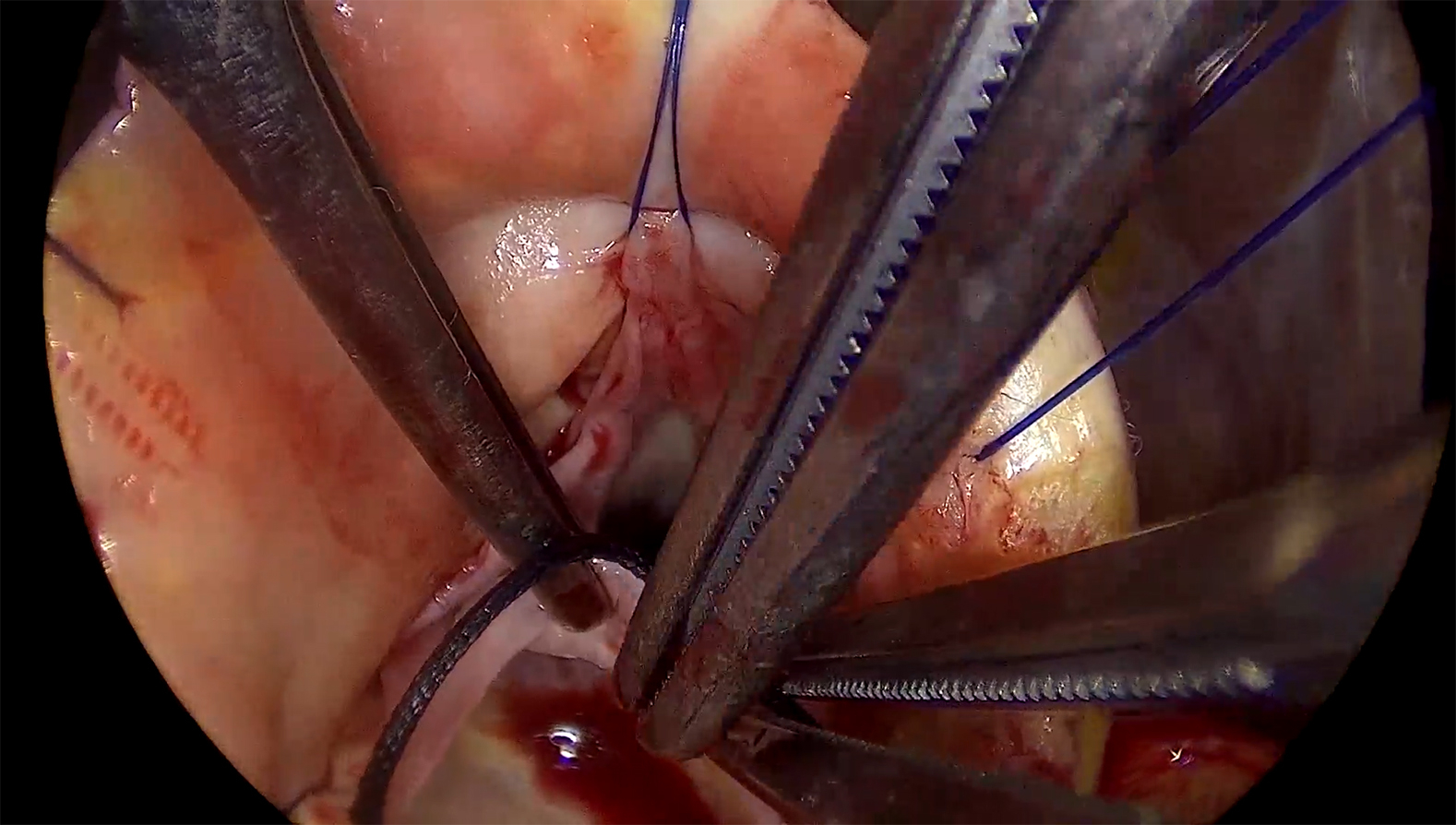

Supplement: Video 6 — Aortic valve repair for leaflet retraction using an augmentation patch of aortic wall. Video available at: https://www.jtcvs.org/article/S2666-2507(23)00111-6/fulltext. [file fx8.jpg]

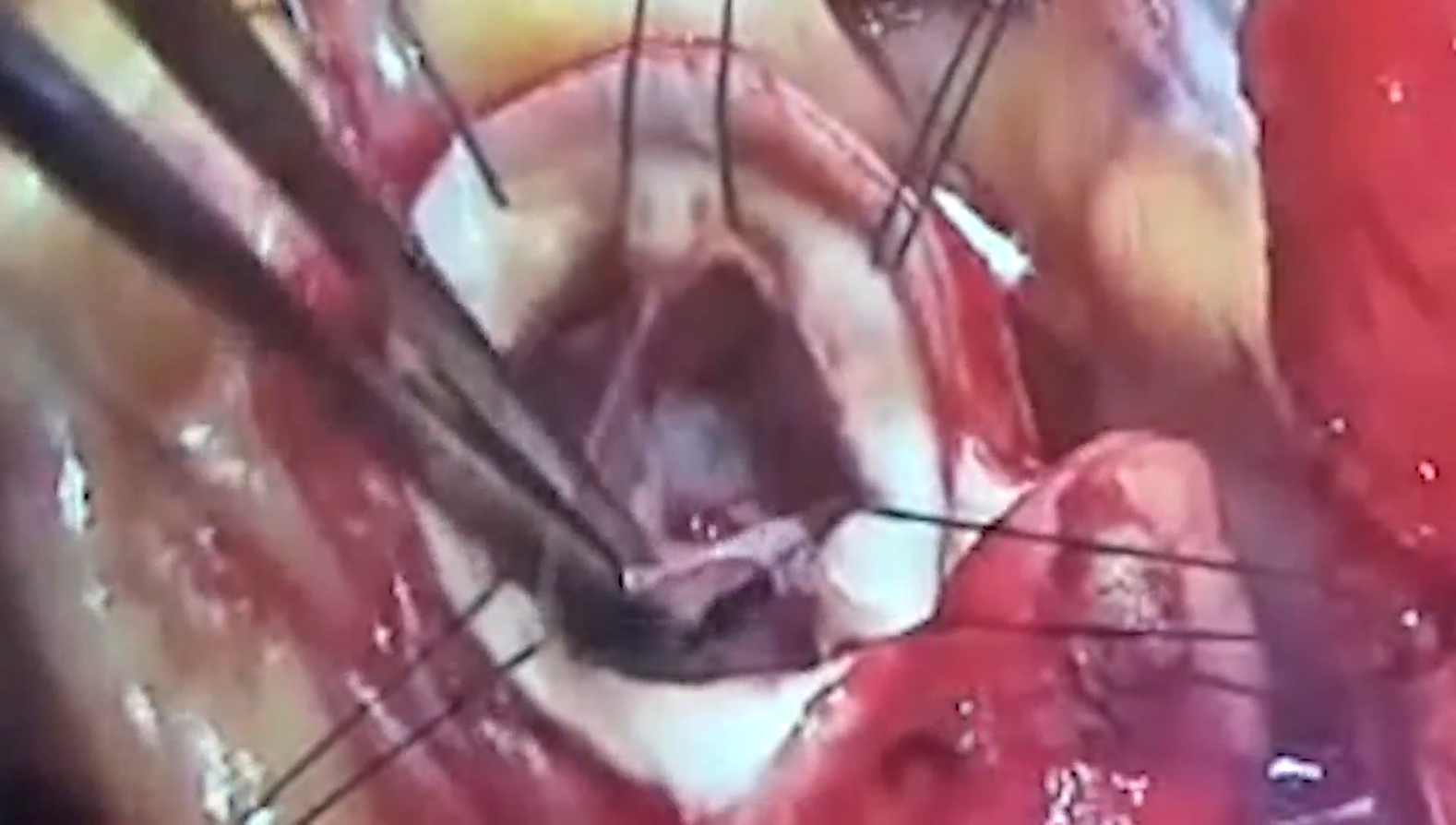

Supplement: Video 7 — Use of an aortic wall patch to repair an endocarditic leaflet hole. Video available at: https://www.jtcvs.org/article/S2666-2507(23)00111-6/fulltext. [file fx9.jpg]
